# Endorsement and phylogenetic analysis of some Fabaceae plants based on DNA barcoding

**DOI:** 10.1007/s11033-022-07574-z

**Published:** 2022-06-02

**Authors:** Nader R. Abdelsalam, Mohamed E. Hasan, Talha Javed, Samar M. A. Rabie, Houssam El-Din M. F. El-Wakeel, Amera F. Zaitoun, Aly Z. Abdelsalam, Hesham M. Aly, Rehab Y. Ghareeb, Alaa A. Hemeida, Adnan Noor Shah

**Affiliations:** 1 Agricultural Botany Department, Faculty of Agriculture, Saba Basha, Alexandria University, Alexandria, 21531 Egypt; 2 Bioinformatics Department, Genetic Engineering and Biotechnology Research Institute, Sadat City University, Sadat City, Egypt; 3 College of Agriculture, Fujian Agriculture and Forestry University, Fuzhou, 350002 China; 4 Genetics Department, Faculty of Agriculture, Ain-Shams University, Ain Shams, Egypt; 5 Department of Forestry and Wood Technology, Horticulture Institute, Agriculture Research Center, Antoniadis Botanical Garden, Alexandria, 21554 Egypt; 6 Plant Protection and Biomolecular Diagnosis Department, Arid Lands Cultivation Research Institute, City of Scientific Research and Technological Applications, Borg El-Arab, Alexandria, 21934 Egypt; 7 Department of Agricultural Engineering, Khwaja Fareed University of Engineering and Information Technology, Rahim Yar Khan, 64200 Punjab Pakistan

**Keywords:** DNA barcoding, Legume tree, MaturaseK gene, Phylogenetic tree

## Abstract

**Background:**

DNA barcoding have been considered as a tool to facilitate species identification based on its simplicity and high-level accuracy in compression to the complexity and subjective biases linked to morphological identification of taxa. MaturaseK gene (*MatK gene)* of the chloroplast is very vital in the plant system which is involved in the group II intron splicing. The main objective of this study is to determine the relative utility of the “*MatK*” chloroplast gene for barcoding in 15 legume as a tool to facilitate species identification based on their simplicity and high-level accuracy linked to morphological identification of taxa.

**Methods and Results:**

*MatK* gene sequences were submitted to GenBank and the accession numbers were obtained with sequence length ranging from 730 to 1545 nucleotides. These DNA sequences were aligned with database sequence using PROMALS server**,** Clustal Omega server and Bioedit program. Maximum likelihood and neighbor-joining algorithms were employed for constructing phylogeny. Overall, these results indicated that the phylogenetic tree analysis and the evolutionary distances of an individual dataset of each species were agreed with a phylogenetic tree of all each other consisting of two clades, the first clade comprising *(Enterolobium contortisiliquum, Albizia lebbek), Acacia saligna*, *Leucaena leucocephala, Dichrostachys Cinerea, (Delonix regia, Parkinsonia aculeata), (Senna surattensis, Cassia fistula, Cassia javanica)* and* Schotia brachypetala* were more closely to each other, respectively. The remaining four species of *Erythrina humeana, (Sophora secundiflora, Dalbergia Sissoo, Tipuana Tipu)* constituted the second clade.

**Conclusion:**

Moreover, their sequences could be successfully utilized in single nucleotide polymorphism or as part of the sequence as DNA fragment analysis utilizing polymerase chain reaction in plant systematic. Therefore, *MatK* gene is considered promising a candidate for DNA barcoding in the plant family Fabaceae and provides a clear relationship between the families.

## Introduction

Fabaceae is considering a large and economically vital family of flowering plants which is usually known as the legume family [1–5]. The Fabaceae family, which has over 490 medicinal plant species 730 genera flowering plants and more than 19,400 species [5–9]. Documentation of the Mediterranean legume crops depending on morphological characteristics has shown tricky and much impossible [10–13]. So, using a DNA-based technique would offer accurate knowledge and facilitate the discrimination of the species. DNA barcoding is new, efficient, quick, low-cost, and standard technique for the fast identification and evaluation of plant and animal species based on DNA sequence from a small fragment of the whole genome in a rapid, accurate [14–18]. DNA barcoding can help to detect species, quick identification of any species that are possibly novel to science and to report the essential ecological and evolutionary questions as a biological instrument [19–25]. DNA barcoding are frequently promoted for their facility to enhance the accessibility of scientific information and new knowledge to the public and non-specialists [26, 27]. Short DNA sequences in DNA barcoding are used to identify the diversity between plant and animal species as molecular markers [28], also, it’s used in an assignment the unknown samples to a taxonomic group, and in-plant biodiversity documentation [29]. DNA barcoding is a potential tool to detect an error in identifying species because similarity-based approaches using DNA barcoding combined with morphology would solve the misidentification based on morphology [30–32]. DNA barcoding could help decrease the limitations of morphological characteristics and hurry up plant and animal species identification since it can detect the organisms at any stage of growth**.** DNA barcodes are designed to create a shared community resource of DNA sequence that is used in the identification or taxonomic classification of any organisms [33]. The usage of DNA barcodes as a tool for plant/ animal identification is based on the establishment of high-value reference databases of sequence [34] which cannot always distinguish between closely related species of land plants or fungi.

The *MatK* gene (1500 bp in length), located inside the intron of the mitochondrially encoded tRNA lysineprovided by HGNC (*trnK)* and codes for maturase as protein, which is involved in Group- II intron splicing, the *trnK* intron of plants encodes the *MatK* open reading frame (ORF). This gene has a high-level rate of substitution [3], a huge proportion of difference at nucleic acid levels at first and second codon place, and low transition and or/transversion ratio and the presence of mutationally conserved regions. Previous data were utilized to identify the molecular markers, which were used to identify the genus/species of these taxa, to provide valuable information for both conventional and molecular studies [11]. The current study, target to evaluate the capacity and the efficiency of *MatK* gene as normal plant barcode marker*;* documentation and identification of 45 plant specimens belonging to 15 species of Fabaceae plant species, and study the useful annotation, homology modeling and sequence analysis to permit an additional efficient use of these sequences between different plant species.

## Materials and methods

### Plant materials

**Fig. 1:**
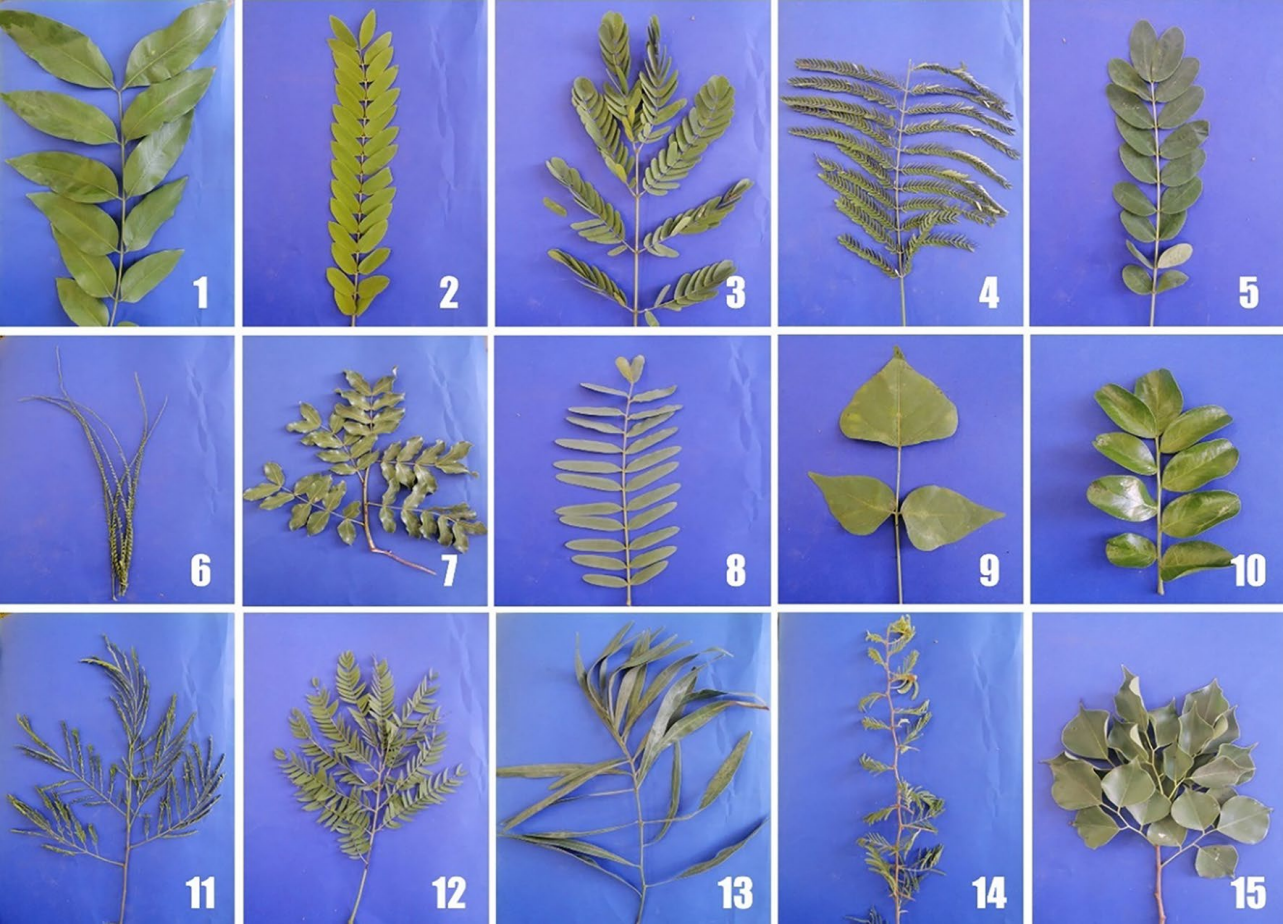
showing difference between leaves in fifteen fabaceae plants (1) *Cassia fistula*, (2) *Cassia javinca,* (3)* Albizia lebbek,* (4) *Delonix regia,* (5) *Senna surattensis,* (6) *Parkinsonia aculeata,* (7) *Schotia brachypetala,* (8)* Tipuana tipu,* (9)* Erythrina humeana, *(10)* Sophora secundiflora,* (11) *Leucaena leucocephala,* (12) *Enterolobium contortisiliquum,* (13) *Dichrostachys cinerea,* (14) *Acacia saligna and* (15) *Dalbergia sissoo*

Forty-five samples (three replicates for each species), which belonged to 15 species found in Fig. 1 were collected from Antoniadis Garden's (N 29" 56′ 55, E 18" 12′ 31), Alexandria, Egypt between July 2019 to January 2020.

### DNA extraction and sequencing of specimens

**Fig. 2:**
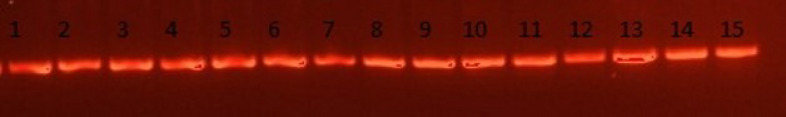
Agarose gel electrophoresis for extracted DNA from samples (1)* Cassia fistula,* (2)* Cassia javinca,* (3) *Albizia lebbek,* (4) *Delonix regia,* (5) *Senna surattensis,* (6) *Parkinsonia aculeata,* (7) *Schotia brachypetala,* (8) *Tipuana tipu,* (9) *Erythrina humeana,* (10) *Sophora secundiflora,* (11)* Leucaena leucocephala,* (12)* Enterolobium contortisiliquum,* (13) *Dichrostachys cinerea,* (14) *Acacia saligna and* (15) *Dalbergia sissoo*

**Fig. 3:**
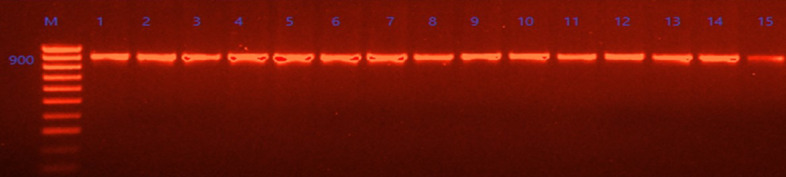
Agarose gel electrophoresis for amplified samples by using the primer *MatK* for the samples (1)* Cassia fistula,* (2)* Cassia javinca,* (3) *Albizia lebbek,* (4) *Delonix regia,* (5) *Senna surattensis,* (6) *Parkinsonia aculeata,* (7) *Schotia brachypetala,* (8) *Tipuana tipu,* (9) *Erythrina humeana,* (10) *Sophora secundiflora,* (11)* Leucaena leucocephala,* (12)* Enterolobium contortisiliquum,* (13) *Dichrostachys cinerea,* (14) *Acacia saligna and* (15) *Dalbergia sissoo*

Total genomic DNA was extracted from fresh leaves tissue by (i-genomic plant DNA extraction Mini kit @ iNtron biotechnology, Berlin, Germany) corresponding to the protocol linked to the Plant Genomic DNA Kit (iNtRON Bio Co., South Korea) as found in Fig. 2. PCR of the *MatK* regions were conducted out in Techne Flexigene PCR Thermal Cycler programmed for 30 cycles as follows: 94 °C/5 min (1 cycle); 94 °C/45 s, 50 °C/45 s, 72 °C/45 s (30 cycles); 72 °C/7 min (1 cycle); 4 °C (infinitive). The designed common primers and reaction conditions of the *MatK* region is F: 5'-CGTACAGTACTTTTGTGTTTACGAG-3' (Tm, 53.9 and GC%, 40), R: 5'-ACCCAGTCCATCTGGAAATCTTGGTTC-3' (Tm, 60.4 and GC%, 48). The PCR products were run on a 1.0% agarose gel utilizing 1X TAE buffer containing 0.5 µg/mL ethidium bromide for electrophoresis of PCR products as found in Fig. 3. PCR products were purified using Mini kit @ iNtron Biotechnology Purification kits before being sequenced exploitation the dideoxynucleotide chain termination method with a DNA sequencer (Applied Biosystems® 3500 and 3500xL Genetic Analyzers) and a BigDye Terminator version 3.1 Cycle Sequencing RR-100 Kit (Applied Biosystems). The sequences were submitted to DDBJ/EMBL/GenBank database. Generic and species data was achieved from the taxonomy database of the National Centre for Biotechnology Information (NCBI).

### Sequence analysis

The sequences results analysis was completed for the one grouped dataset, this set contains all the plant species of Fabaceae for which the sequences are available in GenBank to find the inter-species and inter-generic variation. *Fabaceae* species sequences of *MatK* were retrieved from NCBI in Fasta format. Multiple sequence alignments of the *MatK* gene were conducted from different species applying the PROMALS server [35], Clustal Omega server [36], the BIOEDIT software [37] and MEGA-11 [38] which are offline software that conducts optimal sequence alignment to find the conserved area. The MEGA 11 software has matured to contain a large collection of methods and tools of computational molecular evolution for building timetrees of species, pathogens, and gene families using rapid relaxed-clock methods and estimating divergence times and confidence intervals for node-dating and sequence sampling dates for tip-dating analyses. Comparing to the greatest alignment methods with development for distantly related sequences the “PROMALS” is up to 30% more accurate. Clustal Omega server is a new multiple sequence alignment software that generates alignments between three or more sequences using seeded guide trees and HMM profile-profile methods. The “BIOEDIT” software is a user-friendly biological sequences alignment editor that aims to provide fundamental functions for editing, aligning, manipulating, and analyzing protein sequences.

#### Molecular evolution and phylogenetic analysis

The Neighbor Joining method was used to deduce the evolutionary narrative. Finding the topology and branch length of the tree that will offer the best chance of detecting amino acid sequence in current data is the approach for constructing the phylogenetic tree using maximum likelihood. So, for phylogenetic evaluation Mafft server [39], Clustal Omega server and “MEGA-11” software were applied. MEGA was used to analyze the sequencing data using the neighbor-joining technique and Unweighted Pair Group Mean Average "UPGMA." The “DNADIST” software of “PHYLIP” was used to calculate distances. NJ plot was used to do bootstrapping and decay analysis. MEGA determined parsimony analyses and different clades.

## Results

### DNA extraction and PCR amplification

The quality of the obtained DNA was detected 1% agarose gel electrophoresis. The results indicated that there is no fragmentation was observed in extracted DNA. The quantity of extracted DNA samples was determined by using Nanodrop Spectrophotometer and the concentration ranged from 30 to 50 ng/μl. The extracted DNA was directly used in PCR amplification for the *MatK* gene recorded on fragment in molecular weight (900 bp).

Development in DNA sequencing methods has allowed us to describe the genomes of numerous organisms quickly. Evaluations of the DNA sequences of several species are providing useful knowledge about their taxonomy, gene makeup, and utilization. In the current study using DNA sequence polymorphisms of the chloroplast, *MatK* gene is much more variable than many other genes. From Fifteen plant species belong to different genera of the same family Fabaceae as found in Table 1. In this data we organized a study to contribute to the knowledge of the major evolutionary relationship between the studied plant genus and species (clades) and discussed the application of *MatK* for molecular evolution. The chloroplast *MatK* marker was more useful as DNA markers. The present study included fifteen species from fifteen genera are deposited in GenBank; accession numbers were obtained for the respective plant species with different numbers of conserved domains, segment length and average entropy (Hx) (Table 1).

**Table 1:** Fifteen taxa, their families, and the sequence length (bp), assigned Accession numbers, number of conserved regions and average Entropy (Hx) of each plant were used in this study

Species	Isolate	Length (bp)	Accession number	Number of conserved region	Average Entropy (Hx)
*Acacia saligna*	SR01	714	LC602060	15	0.0000
*Albizia lebbeck*	SR02	788	LC602154	16	0.0000
*Cassia fistula*	SR03	854	LC602263	14	0.0000
*Cassia javanica*	SR04	845	LC603347	14	0.0068
*Dalbergia sissoo*	SR05	763	LC603655	13	0.0354
*Delonix regia*	SR06	864	LC603845	18	0.0000
*Dichrostachys cinerea*	SR07	866	LC603846	22	0.0119
*Enterolobium contortisiliquum*	SR08	856	LC603847	19	0.0204
*Erythrina humeana*	SR09	781	LC604717	13	0.0028
*Leucaena leucocephala*	SR10	820	LC604718	15	0.0203
*Parkinsonia aculeata*	SR11	838	LC605994	15	0.0000
*Schotia brachypetala*	SR12	856	LC604799	21	0.0119
*Senna sulfurea*	SR13	853	LC605995	17	0.0074
*Sophora secundiflora*	SR14	749	LC606468	16	0.0139
*Tipuana tipu*	SR15	787	LC606469	18	0.0353

### Phylogenetic analysis of collected plants

**Fig. 4:**
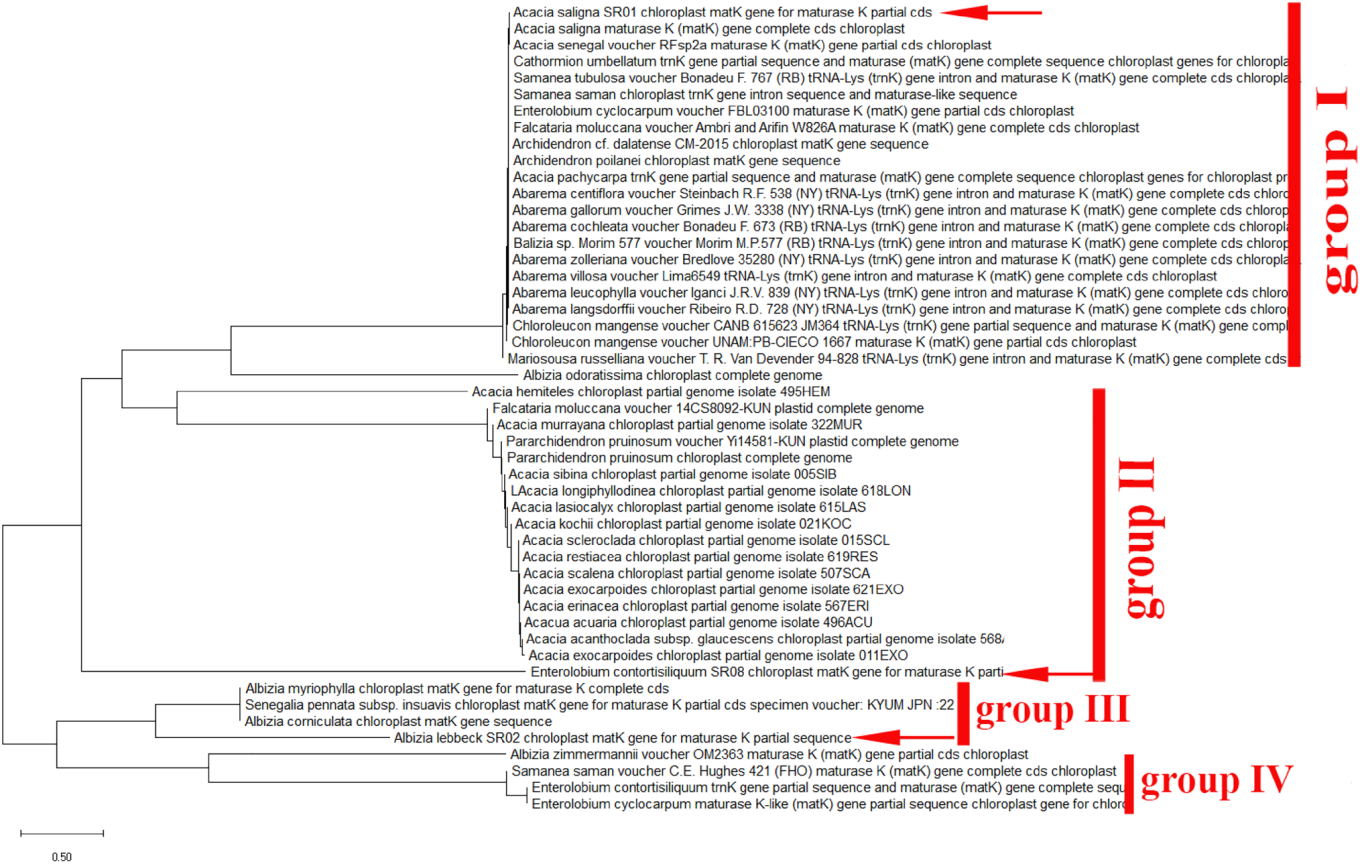
Phylogenetic tree analysis and the evolutionary distances of *Acacia saligna, Albizia lebbeck* and *Enterolobium contortisiliquum* were computed using the Maximum Likelihood technique using nucleotide sequences of the *MatK* gene. This analysis involved 49 nucleotide sequences. Codon positions included were 1st + 2nd + 3rd + Noncoding. All ambiguous positions were removed for each sequence pair (pairwise deletion option). There were a total of 856 positions in the final dataset

Numerous sequence alignments showed that there are varying numbers of “Indels” in the gene *MatK*. Using the neighbor-joining method, UPGMA and maximum likelihood, the evolutionary distances for the 15 plant species were recognized into individual clades. The alignment of *MatK* gene of *Acacia saligna* nucleotide sequences showed 15 conserved regions, 769 variable sites and 571 parsimony sites, the overall mean distance is 2.85 (Table 2). The combined tree showed two groups or cladograms and they are represented as follows: Group I include Acacia saligna was closely related to different species belonging to other genera of the same family (Fabaceae) such as *Enterolobium, Pararchidendron, Archidendron, Samanea, Hydrochorea, Balizia and Abarema* (Fig. 4). Also, Acacia comprising other species were closely arranged but distinguished into different genera such as *Falcataria, Pararchidendron and Lacacia*. In addition, the aligned *MatK* dataset was 793 nucleotide sites long, of which 102 sites were potentially parsimony informative. Consequently, *Enterolobium contortisiliquum* is more closely related to different species of genus Acacia according to phylogenetic analysis using maximum likelihood (Fig. 4). The length of *MatK* varies from 750 bp in *Albizia lebbek* (the smaller length of *MatK* gene for these species is due to incomplete sequencing, which was retrieved from GenBank) to 813 in different genera (E*nterolobium, Acacia, Senegalia, Cojoba, Samanea, Hydrochorea, Balizia and Abarema)*. Maximum likelihood and Neighbor-joining analysis of the dataset resulted in tree with two groups. The clades established in the trees were mainly mixtures of numerous species. Consequently, creating a local barcode database will be useful for a broad range of potential ecological purposes, involving the building of community phylogenies [40]^.^ Group I have three clusters comprising several genera (Albi*zia, Enterolobium, Mariosousa, Archidendron, Samanea, Balizia, and Abarema*). Otherwise, group II has one genera acacia which is the most closely related to our plant Albizia lebbeck according to *MatK* gene partial cds (Fig. 4). The arrangement of *MatK* gene of *Albizia lebbeck* nucleotide sequence revealed 649 varying sites and 359 parsimony sites, the overall mean distance is 2.37 and the estimated Transition/Transversion bias (R) is 0.52 (Table 2, 3).

**Table 2:** The Homogeneity test of substitution patterns between sequences of fifteen studied species

	1	2	3	4	5	6	7	8	9	10	11	12	13	14	15
1. *Acacia saligna SR01*															
2. *Albizia lebbeck SR02*	1.000														
3. *Cassia fistula SR03*	1.000	1.000													
4. *Cassia javanica SR04C*	0.216	0.182	1.000												
5. *Dalbergia sissoo SR05*	0.096	0.106	0.178	0.214											
6. *Delonix regia SR06*	1.000	1.000	1.000	1.000	0.274										
7. *Dichrostachys cinerea SR07*	0.224	0.306	0.186	0.106	1.000	1.000									
8. *Enterolobium contortisiliquum SR08*	1.000	0.182	0.036	0.012	0.028	0.038	0.004								
9. *Erythrina humeana SR09*	0.000	0.000	0.000	0.000	0.000	0.000	0.000	0.000							
10. *Leucaena leucocephala SR10*	1.000	1.000	0.328	0.164	0.060	0.204	0.048	0.258	0.000						
11. *Parkinsonia aculeata SR11*	1.000	1.000	1.000	1.000	0.158	1.000	1.000	0.236	0.000	0.182					
12. *Schotia brachypetala SR12*	1.000	1.000	1.000	1.000	0.282	1.000	1.000	0.156	0.000	1.000	0.274				
13. *Senna sulfurea SR13*	1.000	1.000	0.260	0.030	0.346	1.000	1.000	0.078	0.002	0.146	1.000	0.298			
14. *Sophora secundiflora SR14*	1.000	1.000	1.000	1.000	1.000	1.000	1.000	0.030	0.000	1.000	1.000	1.000	1.000		
15. *Tipuana tipu SR15*	0.274	1.000	1.000	1.000	1.000	1.000	1.000	0.046	0.000	0.184	1.000	1.000	1.000	1.000

**Table 3:** The estimation of evolutionary divergence between fifteen studied species

	1	2	3	4	5	6	7	8	9	10	11	12	13	14
1. *Acacia saligna SR01*														
2. *Albizia lebbeck SR02*	0.0261													
3. *Cassia fistula SR03*	0.0510	0.0626												
4. *Cassia javanica SR04C*	0.0497	0.0586	0.0096											
5. *Dalbergia sissoo SR05*	0.1445	0.1485	0.1293	0.1281										
6. *Delonix regia SR06*	0.0390	0.0484	0.0429	0.0432	0.1329									
7. *Dichrostachys cinerea SR07*	0.0301	0.0401	0.0442	0.0420	0.1382	0.0471								
8. *Enterolobium contortisiliquum SR08*	0.0142	0.0252	0.0687	0.0612	0.1425	0.0580	0.0427							
9. *Erythrina humeana SR09*	0.1818	0.1960	0.1852	0.1834	0.1955	0.1721	0.1783	0.1916						
10. *Leucaena leucocephala SR10*	0.0261	0.0334	0.0583	0.0521	0.1319	0.0419	0.0368	0.0396	0.1814					
11. *Parkinsonia aculeata SR11*	0.0594	0.0865	0.0657	0.0636	0.1533	0.0465	0.0689	0.0790	0.1995	0.0779				
12. *Schotia brachypetala SR12*	0.1159	0.1282	0.0998	0.0947	0.1594	0.1169	0.1166	0.1257	0.2082	0.1342	0.1267			
13. *Senna sulfurea SR13*	0.0666	0.0788	0.0278	0.0267	0.1431	0.0605	0.0529	0.0778	0.1959	0.0763	0.0823	0.1157		
14. *Sophora secundiflora SR14*	0.0978	0.0967	0.0931	0.0872	0.0903	0.0900	0.0981	0.1043	0.1465	0.1040	0.0946	0.1184	0.1059	
15. *Tipuana tipu SR15*	0.1241	0.1356	0.1187	0.1192	0.0641	0.1186	0.1203	0.1342	0.1813	0.1167	0.1490	0.1504	0.1348	0.0725

**Fig. 5:**
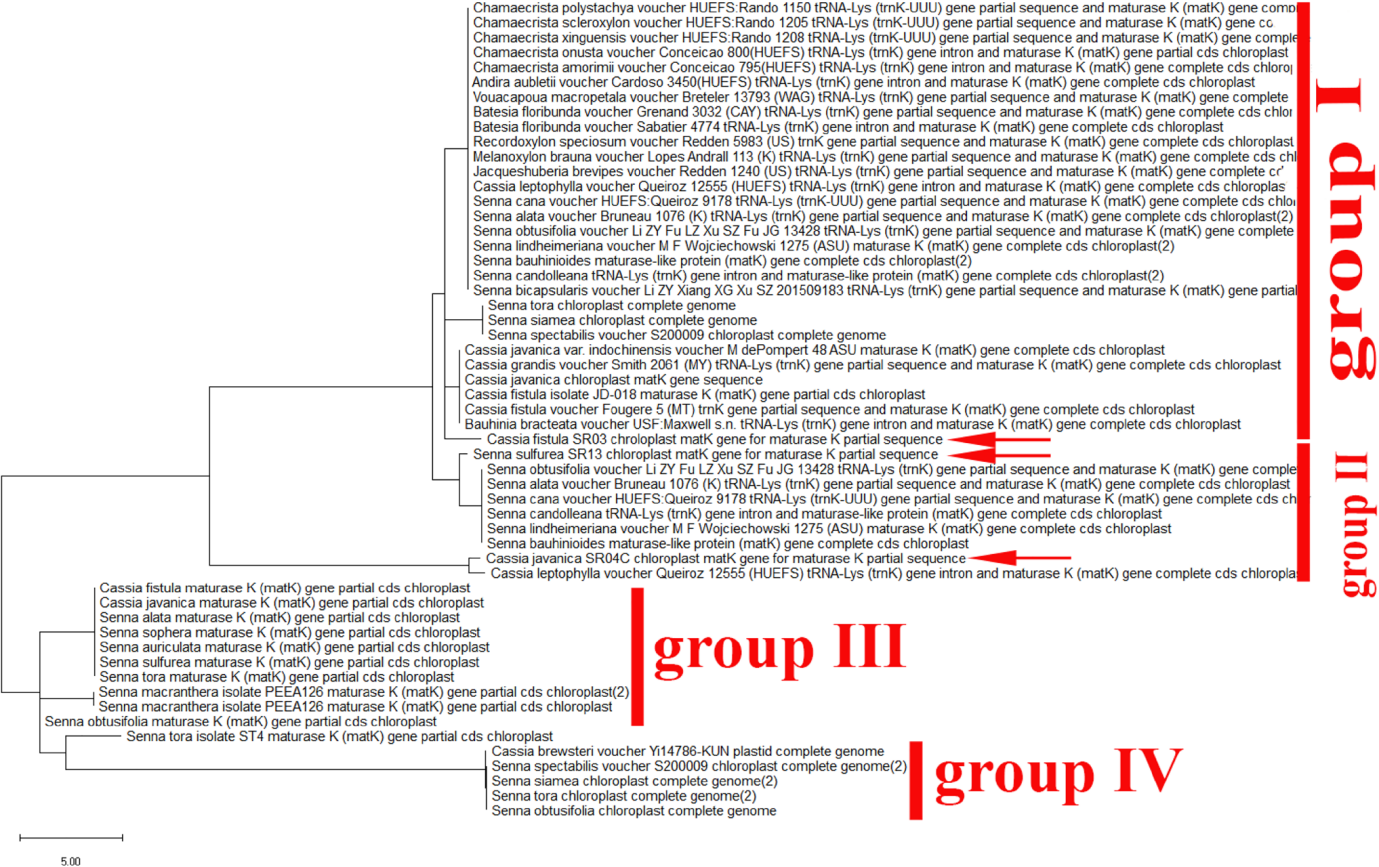
Phylogenetic tree analysis and the evolutionary distances of *Cassia fistula, Cassia javanica and Senna surattensis* using the Neighbor-Joining technique using nucleotide sequences of the *MatK* gene. The tree is drawn to scale, with branch lengths measured in the number of substitutions per site with the highest log likelihood (− 16,447.32). This analysis involved 55 nucleotide sequences and there were a total of 854 positions in the final dataset

Furthermore, depending on the phylogenetic analysis, the two genera Cassia and Senna with different species are closely related and more highly similar than any other studies species (Fig. 5). The phylogeny tree was created using the neighbor-joining approach and the evolutionary distances were calculated employing the maximum composite likelihood approach. The combined trees showed that there are two groups, and they are as follows: Group I consisted of five clades representing different genera with different species such as (*Chamaecrista, Senna, Erytherophleum, Arapatiell and Dinizia*). Group II showed two branches: each one with many sub-branches containing five clades with different species of the genus Senna. According to *MatK* gene sequence, the collected plants (*Cassia fistula and Cassia javanica*) revealed a high percentage of identity with different 7 species of genus senna having the same clade (Fig. 5). Also, they are closely related to other different species of *Erytherophleum, Arapatiell*. On another hand, the sequence of *MatK* gene of collected Senna surattensis species has a high degree of similarity with many species in different genera in Fabaceae (Fig. 5), and consequently, this species is used as a template to estimate the similarity between different species in Fabaceae family.

**Fig. 6:**
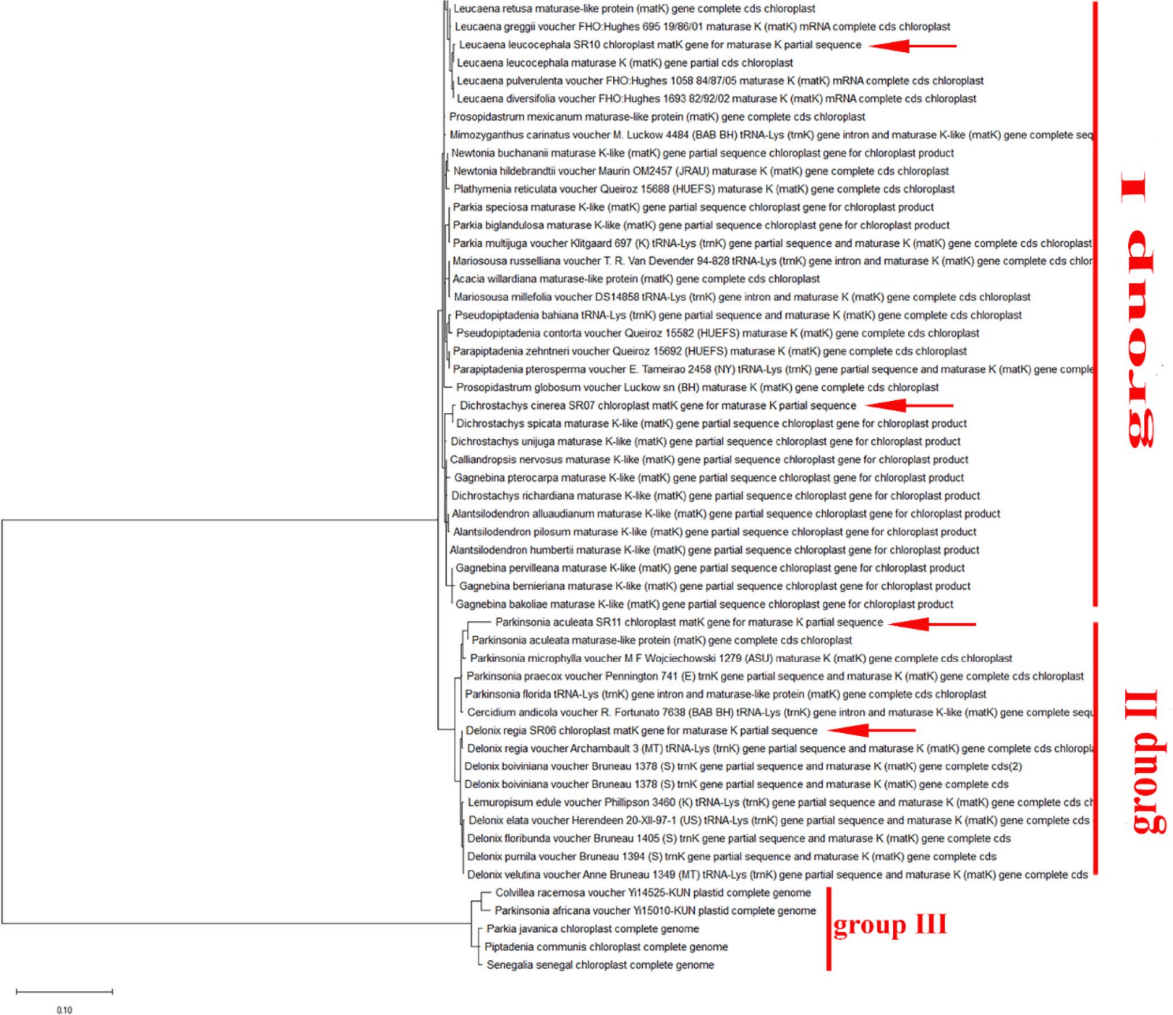
Phylogenetic tree analysis and the evolutionary distances of *Leucaena leucocephala, Dichrostachys Cinerea, Delonix regia* and *parkinsonia aculeata* using the Neighbor-Joining technique using nucleotide sequences of the *MatK* gene. This analysis involved 73 nucleotide sequences. All ambiguous positions were removed for each sequence pair (pairwise deletion option). There were a total of 924 positions in the final dataset

Nevertheless, the *Delonix regia* is the more studied species having a good similarity to different species of different genera of the Fabaceae family. Polymorphism obtained from the DNA sequence indels or replacements of the *MatK* gene indicated that *Delonix regia, Umtiza listeriana, Diptychandra aurantiaca, Moldenhawera blanchetiana, Schizolobium parahyba, Tachigali costaricensis, Arapatiella psilophylla and Parkinsonia Africana* were evolved from a Common ancestor (Fig. 6). In addition, *Dichrostachys cinerea* is closely related to different species of genera *Leucaena, Senegalia, Falcataria and prosopis* (Fig. 6). Furthermore, applying the same incremental method of informative sites starting at the 5/-end of the *MatK* gene, completely different results were found. The consensus tree of 15 most parsimonious trees demonstrated unresolved clades until 250 informative sites. At that point, 1 highly parsimonious tree was created, which was congruent with the topology of the stable tree achieved from the 3/-end. To recognize the greatest DNA barcode marker for species documentation and traceability, the value of genetic divergence for all the confirmed loci were calculated in each analyzed group at dissimilar taxonomic level and by considering only fresh morphologically identified samples. Results indicated the species of *Delonix regia, Parkinsonia aculeata and Leucaena leucocephala* are more like other species of the same genus and less similar to species of other genera of the Fabaceae family (Fig. 6). This reflected that the *Parkinsonia aculeata* was closely related and in the same clade with *Schizolobium parahyba, Diptychandra aurantiaca, Delonix regia, Conzattia multiflora and Colvillea racemose* (Fig. 6).

**Fig. 7:**
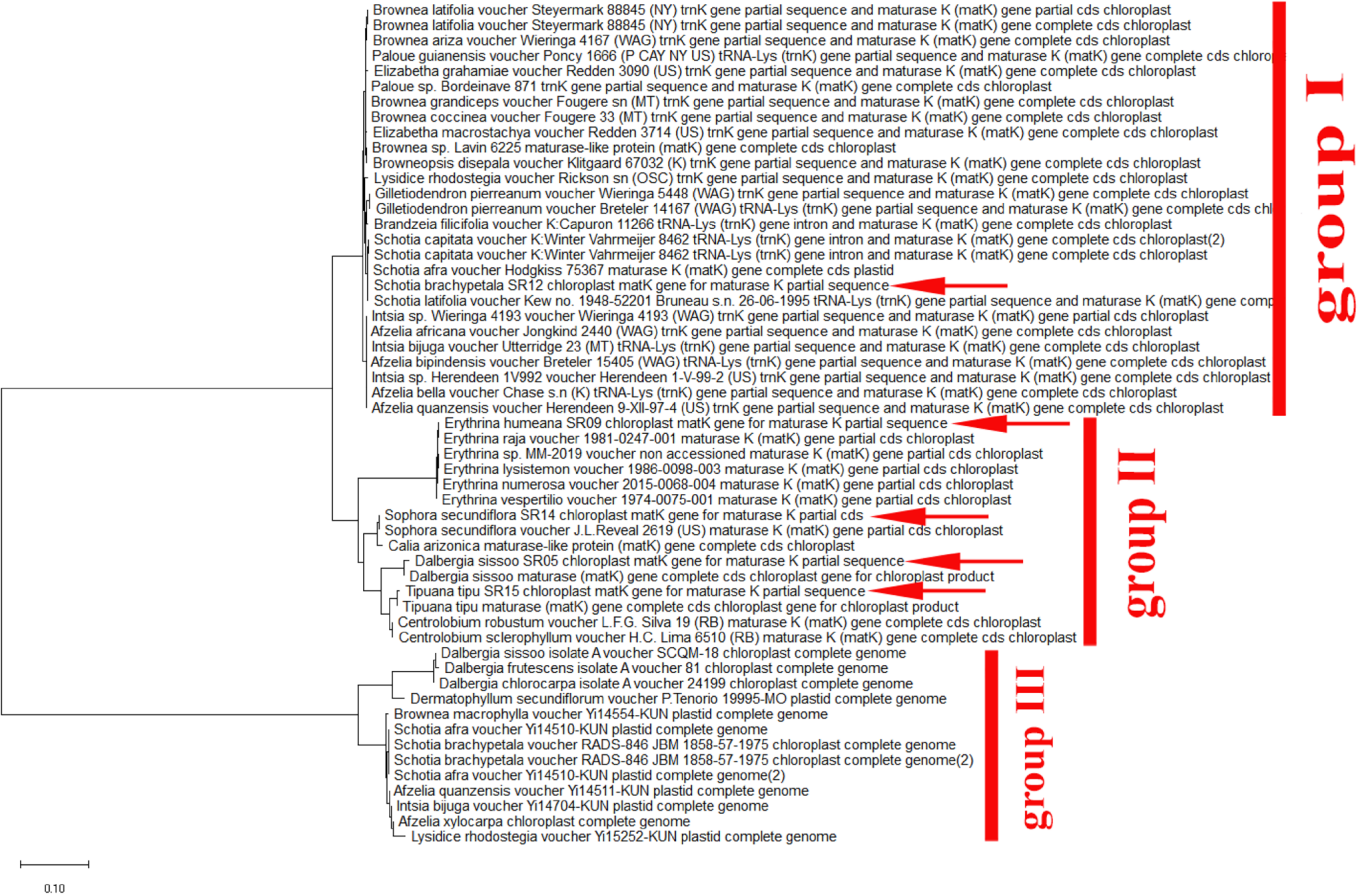
Phylogenetic tree analysis and the evolutionary distances of Dalbergia Sissoo, Erythrina humeana, Schotia brachypetala, Sophora secundiflora and Tipuana Tipu using using the Maximum Likelihood method. The tree is drawn to scale, with branch lengths measured in the number of substitutions per site with the highest log likelihood (− 4725.80). This analysis involved 55 nucleotide sequences. There were a total of 902 positions in the final dataset

The current sequences showed little variations in the percentage of guanine plus cytosine content (% G + C) related to that in the sequences of *MatK*. In case of *MatK*, the nucleotide structure was biased toward the guanine and cytosine content (G + C) with frequencies were 30.4 to 34.8%, respectively. The NJ, ML, and MP analyses all resulted in comparable trees in each of the data sets. There are often variations between the trees from the various analyses involving non-resolution (polytomies). Analyses carried out on samples belonging to *Parkinsonia aculeata, Schotia brachypetala, Sophora secundiflora and Tipuana Tipu* indicated that the sequences divergences of marker *MatK* were clearly distinguished from other species of Fabaceae. Figures 7 showed phylogenetic clusters constructed using ML and NJ; The difference observed in *MatK* does separate several species; however, there is a wide range of intra- specific and inter-specific variation. Furthermore, On the Neighbor-Joining Phylogram, the Schotia group is a sister taxon to the Macrolobium group and this observation is found in 50% of the most clade in this cladistic analysis (Fig. 7).

The last two members i.e., *Sophora secundiflora* and *Tipuana Tipu* produced an independent clade and confirmed the ambiguous position relative to the other genera of Fabaceae based on the combined cladistic analysis data from chloroplast DNA restriction sites and morphology. Sophora secundiflora shared a common ancestor with *Angylocalyx braunii, Zollernia splendens, Ormosia xylocarpa and Dermatophyllum secundiflorum. Also, Tipuana Tipu* is in the same clade with different species of two genera *Centrolobium and Pterocarpus* (Fig. 7). Additionally, highly Fabaceae species in the current research were detected to have a unique sequence in the *MatK* gene. These results will offer a valuable way to authenticate various *MatK* species. *MatK* sequence created in this analysis will be applied to construct reference sequence libraries, and the sequences extracted from samples with particular identity classifications will be utilized to search the database.

**Fig. 8:**
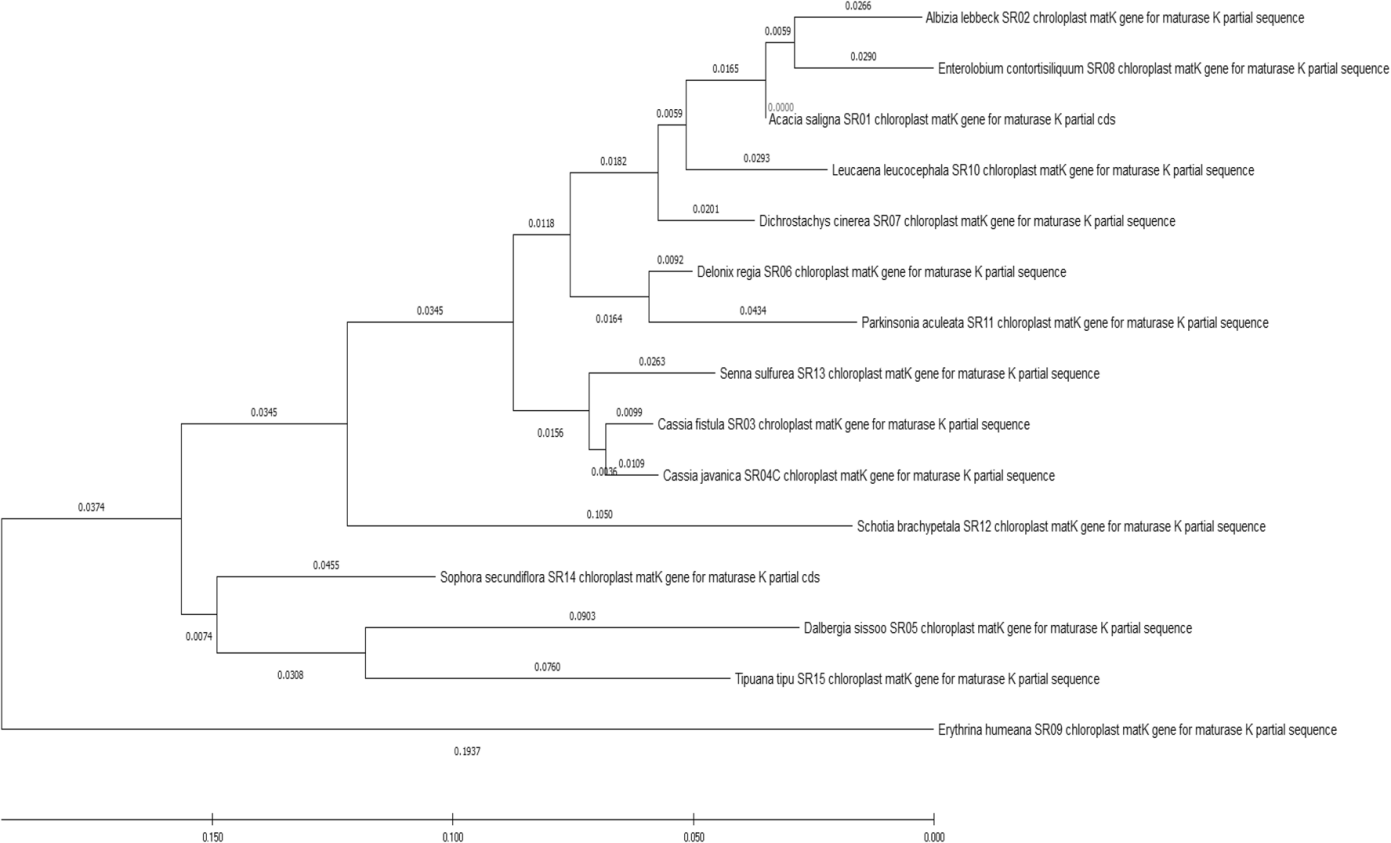
Evolutionary analysis of legume tree species grown in Egypt in this study using *MatK* gene by Maximum Likelihood method

Lastly, utilizing BLAST1 and the closest genetic distance approach, we will be able to define the species identities of the query sequences based on these data. In the dataset of *MatK*, the nearest genetic distance approach achieved 99.68% to 96.45% identification accomplishment rates at the species level for “BLAST1” and distance discrimination methodology, respectively, with no equivocal identification at the genus level. The planned barcoding portion of *MatK* is about 760 base pairs in Fabaceae. The phylogenetic tree (Fig. 8) consists of two clades, the first clade comprising (*Enterolobium contortisiliquum, Albizia lebbek), Acacia saligna, Leucaena leucocephala, Dichrostachys Cinerea, (Delonix regia, Parkinsonia aculeata), (Senna surattensis, Cassia fistula, Cassia javanica) and Schotia brachypetala* were more closely to each other, respectively. The other four species of *Erythrina humeana with Sophora secundiflora and (Dalbergia Sissoo, Tipuana Tipu*) constituted the second clade.

## Discussion

Because plant genomes include several copies of *MatK* sequences, it's unclear if the sequence obtained by PCR will be balanced and representative [41]. As a result, we suggest *MatK* as a potential barcode sequence in the Fabaceae family, as well as a wider range of plant species. Utilizing *MatK* as a DNA barcode would extend our knowledge of phylogenetics and population genetics in Fabaceae species as reviewed by [42–45]. We also recommend that *MatK* be used as a DNA barcode sequence to overcome difficulties in Fabaceae genus and species categorization [44, 46]. *MatK might serve as a starting point for quality control and assurance of plant materials utilized in research, manufacturing, customs, and forensics*.

The *MatK* was discovered to be a necessarily variable DNA region between Fabaceae species as determined by genetic divergences, and it demonstrated a greater potential of effective discrimination. *MatK* can be a powerful taxonomic marker for identifying species and resolving taxonomic issues [30, 32, 41]. For instance, the *MatK* sequence of Enterolobium contortisiliquum is highly like Albizia lebbek, so our results indicate that in the genus Cassia, in which the species were poorly graded, *MatK* was still able to distinguish among some confusing species[47]. The evolutionary distances for the 15 plant species that were separated into distinct clades were analyzed using the maximum likelihood and neighbor-joining methods, which discriminated most of the species better than previous techniques [48].

The identification by *MatK* region paired with morphological recognition 100% to species (Fig. 1) level; for the set of plants studied, it appears to be an accurate approximation of species identification using this one locus. Short sequence, universality, and unique identifiers are three features of a common barcode [48, 49]. According to our results of sequence length and composition of *MatK* barcode gene for the 15 plant species, *MatK* regions have high rate of nucleotide substitutions as showed by [50] or the locus remodeling ring [51]. Alternate primer sequences may increase the success rate of *MatK* amplification for some of the current taxa, making it a barcoding locus. The species in which the *MatK* region is amplified, however, had wide taxonomic coverage in the Fabaceae family, indicating that the locus' conserved sequence is notable.

Consequently, the partial amplification sequence of *MatK* was further utilized to investigate the evolutionary linkage of the selected plants. The evolutionary distances between the 15 plant species were divided into two clades using the neighbor-joining approach., the first clade comprising *(Enterolobium contortisiliquum, Albizia lebbek), Acacia saligna*, *Leucaena leucocephala, Dichrostachys Cinerea, (Delonix regia, Parkinsonia aculeata), (Senna surattensis, Cassia fistula, Cassia javanica)* and* Schotia brachypetala* which were more closely to each other, respectively. The remaining four species of *Erythrina humeana, (Sophora secundiflora, Dalbergia Sissoo, Tipuana Tipu)* constituted the second clade. The results are encouraging, which give a backbone of knowledge in the data set. As additional species become accessible, more research for species resolution of a genus may be undertaken.

## Conclusion

During the current study, DNA barcoding using *MatK* chloroplast gene was applied in fifteen legume trees by both single region and multiregional approaches. The obtained chloroplast gene sequences were submitted to GenBank, and fifteen accession numbers were recorded as LC602060, LC602154, LC602263, LC603347, LC603655, LC603845, LC603846, LC603847, LC604717, LC604718, LC605994, LC604799, LC605995, LC606468, LC606469) with length ranging from 730 to 1545 nucleotides. The current results indicated that the phylogenetic tree analysis and the evolutionary distances of an individual dataset of each studied species were agreed with a phylogenetic tree of all each other consisting of two clades, the first clade comprising *(Enterolobium contortisiliquum, Albizia lebbek), A. saligna*, *Leucaena leucocephala, Dichrostachys Cinerea, (Delonix regia, Parkinsonia aculeata), (Senna surattensis, C. fistula, C. javanica)* and* Schotia brachypetala* were more closely to each other, respectively. The remaining four species of *Erythrina humeana, (Sophora secundiflora, Dalbergia Sissoo, Tipuana Tipu)* constituted the second clade. Finally*, it could be concluded that, MatK* gene is considered promising a candidate for DNA barcoding in plant family Fabaceae and providing a clear relationship between the families.
